# Proactive personality, burnout, and teaching enjoyment: exploring relationships in Chinese English teachers

**DOI:** 10.3389/fpsyg.2024.1351313

**Published:** 2024-11-29

**Authors:** Hu Chunyan, Liao Ying

**Affiliations:** ^1^Primary Education Department, Chongqing Preschool Education College, Chongqing, China; ^2^School of Foreign Languages, Southwest Petroleum University, Chengdu, China

**Keywords:** proactive personality, burnout, teaching enjoyment, English as a foreign language, Chinese educators

## Abstract

**Introduction:**

This study examines the relationships between proactive personality, burnout, and teaching enjoyment among Chinese English as a Foreign Language (EFL) teachers. The research aims to clarify how proactive personality traits relate to teaching enjoyment and burnout and to explore their interactions in the context of EFL instruction.

**Methods:**

The study included a sample of 420 Chinese EFL teachers, and Structural Equation Modeling (SEM) was used to assess the relationships among proactive personality, burnout, and teaching enjoyment. Data underwent detailed statistical analysis to identify both direct and mediating effects within the proposed model.

**Results:**

The findings from the SEM analysis revealed significant direct relationships between proactive personality, burnout, and teaching enjoyment among Chinese EFL educators. Proactive personality was associated with a decrease in burnout (*β* = −0.366, *SE* = 0.159, *p* < 0.001). For every unit increase in proactive personality, teachers reported experiencing higher levels of teaching enjoyment (*β* = 0.487, *SE* = 0.176, *p* < 0.001). Teaching enjoyment, in turn, was a significant mediator in the relationship between proactive personality and burnout (*β* = −0.249, *SE* = 0.102, *p* < 0.001). These findings suggest that proactive personality is positively associated with teaching enjoyment, which, in turn, may relate to lower levels of burnout among EFL teachers.

**Discussion:**

This study highlights the importance of proactive personality traits in enhancing teaching enjoyment and reducing burnout among Chinese EFL teachers. The findings emphasize how proactive tendencies contribute to teachers’ well-being, professional satisfaction, and, ultimately, the quality of EFL instruction. These results suggest practical applications, such as developing interventions that encourage proactive traits and increase teaching enjoyment among EFL educators, which may improve the educational experience for EFL students.

## Introduction

The teaching profession is essential to society, influencing the knowledge, values, and character of future generations. Educators carry substantial responsibilities, motivated by commitment and passion. However, teaching also brings challenges, with high job-related stress and burnout remaining significant concerns (Chang, 2009; Chen, 2023; Chen et al., 2023; Shen et al., 2015). Burnout not only affects teachers’ well-being but can also reduce the quality of education and negatively impact students (Brady et al., 2023; Cheng et al., 2023; Oliveira et al., 2021; Skaalvik and Skaalvik, 2017).

Identifying factors that enhance teacher motivation and well-being is essential for creating a sustainable educational environment. Self-determination theory (SDT) provides a useful framework for understanding these influences. SDT distinguishes between intrinsic motivation—stemming from genuine interest and enjoyment—and extrinsic motivation, which is driven by external incentives (Deci and Ryan, 2000; Ryan and Deci, 2000). Intrinsic motivation is linked to greater persistence, creativity, and well-being. Encouraging intrinsic motivation in educators may positively affect teaching enjoyment and help mitigate burnout.

Research increasingly explores how individual differences and personal traits shape the experience of teacher burnout, given its complex nature (Chen, 2023; Chen et al., 2023; Madigan and Kim, 2021). Proactive personality, a trait marked by self-initiative at work, is one such characteristic attracting scholarly attention (Bateman and Crant, 1993; Fuller and Marler, 2009; Wang et al., 2021). Educators with a proactive disposition tend to take initiative, pursue professional development, and actively engage in problem-solving (Wang et al., 2021). They are more inclined to set goals, seek achievements, and shape their work environments to better align with their aspirations (Dai and Wang, 2023; Grant and Ashford, 2008; Maan et al., 2020). The potential link between proactive personality and teacher burnout is intriguing, suggesting that proactive teachers may display greater resilience and adaptability in managing the demands and challenges of teaching (Kong et al., 2021; Pietarinen et al., 2021). However, despite the appeal of this connection, the mechanisms through which proactive personality affects burnout remain largely underexamined.

Teaching enjoyment is a key emotional factor influencing educators’ job satisfaction and well-being (Ergün and Dewaele, 2021; Frenzel et al., 2016; Skaalvik and Skaalvik, 2017). Educators who find joy in their work often report higher satisfaction, motivation, and a stronger emotional connection to their roles (Frondozo et al., 2020; Schaufeli et al., 2006). Yet, the factors contributing to teaching enjoyment and its potential role in mitigating burnout warrant further investigation.

This study examines how proactive personality influences teacher burnout, focusing on the mediating role of teaching enjoyment. We hypothesize that proactive personality, defined by an engaged and self-initiated work approach, enhances teaching enjoyment, which in turn may help mitigate burnout. Insights from this research hold practical implications for educators, institutions, and policymakers, offering guidance for strategies in teacher development, recruitment, and support that could improve educators’ well-being and educational outcomes. By exploring these relationships, the study contributes to ongoing efforts to improve teachers’ working conditions—a goal with direct benefits for both students and the broader educational system. While cross-sectional studies provide valuable information about associations between variables at a single time point, they do not establish causality. Thus, while this study identifies correlations among proactive personality, teaching enjoyment, and burnout in Chinese EFL teachers, future longitudinal studies are needed to confirm potential causal pathways.

## Review of the literature

### Teacher burnout

Teacher burnout, as defined by Iancu et al. (2018), is a pervasive and intricate psychological response arising from persistent occupational stressors within the field of education. This phenomenon is characterized by a triad of emotional exhaustion, depersonalization, and a diminished sense of personal accomplishment (Cano-García et al., 2005; Maslach et al., 1997). Emotional exhaustion involves feelings of being emotionally drained and depleted, often accompanied by physical and psychological fatigue. Depersonalization manifests as negative, cynical attitudes and behaviors toward students and colleagues, leading to emotional detachment. Reduced personal accomplishment denotes a decline in competence and achievement within the educator’s role (Maslach and Leiter, 2008). Burnout, considered a syndrome, significantly impacts individuals both emotionally and physically, ultimately impairing their effectiveness in the workplace (Maslach et al., 2001; Skaalvik and Skaalvik, 2007).

The concept of burnout initially surfaced in the early 1970s when Freudenberger (1974) introduced it within human service professions. Since then, it has been extensively studied and adapted to various occupational settings, particularly within education. Maslach and Jackson (1981) specifically conceptualized teacher burnout in the late 1970s, leading to the development of widely utilized assessment tools like the Maslach Burnout Inventory (MBI) to measure teacher burnout (Maslach et al., 1997).

The significance of teacher burnout within the educational landscape cannot be overstated, given its multifaceted impact on educators, students, and the broader educational system. Grayson and Alvarez (2008) highlighted its profound negative effects on educators’ physical and mental health, job satisfaction, and retention in the profession. Teachers experiencing burnout face increased susceptibility to stress-related health issues, such as depression and anxiety (Hakanen and Schaufeli, 2012; Zhao et al., 2023), contributing to higher rates of considering leaving the profession (Ingersoll and Strong, 2011). The resulting high turnover not only disrupts educational continuity but also incurs substantial financial and organizational costs for schools and districts (Molero Jurado et al., 2019). Moreover, teacher burnout detrimentally influences students’ learning and well-being. Emotionally exhausted and detached educators provide less effective instruction, leading to lower academic achievement and decreased student engagement (Johnson et al., 2012). Additionally, Saloviita and Pakarinen (2021) pointed out that students can perceive and be adversely affected by teachers’ emotional and attitudinal shifts, creating a less supportive and motivating learning environment.

At a systemic level, high levels of teacher burnout pose considerable challenges for the educational system (Stein et al., 2024). Schools and districts struggle to attract and retain talented educators, resulting in shortages of qualified teachers (Grayson and Alvarez, 2008). The associated costs of recruiting and training replacements for those leaving due to burnout are substantial (Ingersoll and May, 2012; Yang and Ling, 2023), potentially compromising the overall quality and efficacy of educational institutions (Nagy, 2017). Recognizing the gravity of this issue, educational institutions and policymakers have increasingly focused on implementing preventive and supportive interventions (Hakanen et al., 2006). These interventions encompass strategies aimed at reducing stressors, improving teacher well-being, and enhancing job satisfaction. Understanding contributing factors, including individual characteristics like proactive personality, can inform the development of tailored interventions that target specific risk factors and enhance teacher resilience (Kong et al., 2021; Pressley, 2021).

Continued research efforts aimed at understanding the antecedents and consequences of teacher burnout are essential (Hultell et al., 2013; Li, 2023). Valid assessment tools like the Maslach Burnout Inventory for Educators (MBI-Ed) have facilitated consistent measurement, enabling researchers to delve into various contributing factors, both individual and contextual, in the progression of burnout (Hakanen and Schaufeli, 2012; Skaalvik and Skaalvik, 2010).

In summary, teacher burnout significantly impacts educators, students, and the educational system, emphasizing the importance of its understanding and assessment. Its conceptualization has laid the groundwork for research and practical interventions aimed at preserving teacher well-being, improving education quality, and addressing challenges within the teaching profession. Understanding the causes and consequences of teacher burnout remains important in promoting educator health, effectiveness, and the sustainability of the education system.

### Proactive personality

Proactive personality, a construct encapsulating an individual’s propensity for proactive behaviors across various life domains, especially in the workplace, has garnered substantial attention in research (Alikaj et al., 2021; Crant, 2000; Spitzmuller et al., 2015). This disposition characterizes individuals as proactive and self-starting, inclined to take initiative in problem-solving and identifying opportunities (Din et al., 2023; Fuller and Marler, 2009; Grant and Ashford, 2008). Rooted in trait theory within personality psychology, proactive personality reflects an enduring inclination to actively influence one’s environment rather than passively reacting to external circumstances (Bateman and Crant, 1993; Costa and McCrae, 1992).

Significantly, proactive personality extends its influence to individual and organizational outcomes, especially within the workplace (Mubarak et al., 2021). Firstly, it aligns with higher job performance by fostering increased productivity and efficiency (Bateman and Crant, 1993; Crant, 2000). Individuals exhibiting proactive behaviors contribute to achieving work-related goals through their willingness to take on additional responsibilities and suggest improvements (Doğanülkü and Korkmaz, 2023). Moreover, proactive personality is intricately linked to fostering innovation and creativity within organizational settings (Frese and Fay, 2001). Proactive individuals are more likely to generate novel ideas and solutions, thereby aiding problem-solving and organizational development efforts (Köksal et al., 2023).

Career success also aligns with proactive personality, as individuals inclined toward proactivity tend to identify opportunities and actively pursue them, resulting in career advancement and the realization of long-term career goals (Seibert et al., 1999). Work engagement, another facet influenced by proactive personality, is positively associated with the trait. Engaged employees exhibit enthusiasm, energy, and dedication in their tasks, attributes that proactive individuals often possess (Bakker and Leiter, 2017; Xanthopoulou et al., 2009). Proactive individuals actively seek ways to enhance their job roles and responsibilities, aligning them more closely with their work. Additionally, proactive personality contributes to adaptability and resilience in the face of change and uncertainty (Bindl et al., 2012; Fuller and Marler, 2009). Proactive individuals are more likely to perceive change as an opportunity for growth, enabling them to navigate organizational transitions more effectively.

Furthermore, the traits associated with proactive personality often overlap with leadership competencies, thereby positively correlating proactive personality with leadership effectiveness (Harding and Rouse, 2007; Pan et al., 2018). While proactive personality offers numerous advantages, its impact can be contingent on contextual factors such as organizational culture and leadership styles (Parker et al., 2019). Excessive proactivity, if not well-directed, may lead to stress and negative outcomes (Crant, 2000; Crant et al., 2016).

Individuals who are proactive possess a repertoire of personal assets that contribute to their well-being and potentially mitigate burnout. These assets extend beyond resilience, self-confidence, and flexible coping mechanisms to encompass characteristics like perfectionistic strivings (e.g., striving for flawlessness) and specific Big Five personality traits (e.g., conscientiousness, agreeableness) (Samfira and Paloş, 2021). By actively leveraging these personal resources, proactive individuals can work to prevent resource depletion, including emotional exhaustion, depersonalization, and diminished personal achievement, which are hallmarks of burnout (Zhang et al., 2022). A growing body of research delves into the intricate relationship between proactive personality and burnout, revealing its potential impact on educators’ well-being. Proactive personality, as characterized by initiative, self-directed action, and a relentless focus on improvement (Pan et al., 2021; Dai and Wang, 2023), translates into the classroom setting for Chinese English teachers. These individuals actively seek solutions to challenges, readily take the initiative to implement new teaching methods, and demonstrate a strong desire for continuous improvement. These proactive tendencies are hypothesized to influence teachers’ professional experiences in several ways.

Firstly, research by Zhang et al. (2022) suggests that proactive personality’s association with burnout is mediated by job satisfaction. This implies that proactive individuals are better equipped to cultivate job satisfaction, ultimately reducing burnout. Similarly, Kong et al. (2021) found an inverse relationship between proactive personality and academic burnout among nursing students, mediated by professional self-efficacy. This research suggests that proactive behaviors might foster self-efficacy, which in turn acts as a buffer against burnout.

Furthermore, Pietarinen et al. (2021) emphasize the efficacy of proactive measures in mitigating teacher burnout at both individual and school levels. This aligns with the notion that proactive teachers can identify and address challenges before they escalate, potentially reducing burnout for themselves and colleagues. Additionally, Li et al. (2017) highlight that the link between proactive personality and job satisfaction among educators is mediated by self-efficacy and work engagement. Proactive individuals might leverage their initiative to cultivate self-efficacy and work engagement, leading to greater job satisfaction and potentially buffering against burnout. Finally, Wang et al. (2021) provide further support for the positive association between proactive personality and career adaptability and growth potential, which aligns with the conservation of resources theory. Proactive teachers might be better equipped to identify and acquire resources to manage stress and navigate challenges, aligning with the theory’s focus on resource conservation.

However, while proactive personality offers various professional advantages, understanding its limitations within diverse work contexts is essential. Contextual factors may influence how proactive behaviors are received, and excessive proactivity can sometimes lead to increased stress or unintended negative outcomes. Studies indicate that the drive for self-initiated actions can create heightened expectations and added pressure, especially in high-demand or conflicting work environments. Therefore, while proactive traits can support innovation, productivity, and career growth, they must be applied thoughtfully and with attention to context to maximize their benefits and mitigate possible drawbacks.

### Teaching enjoyment

Teaching enjoyment, defined as the amalgamation of affirmative emotions, contentment, and fulfillment teachers experience within their profession, encapsulates an intrinsic sense of happiness, purpose, and gratification derived from their instructional endeavors (Derakhshan et al., 2022; Skaalvik and Skaalvik, 2017; Zhang et al., 2023a). Originating from psychology’s exploration of emotions and job satisfaction, this construct gained recognition within educational contexts as researchers delved deeper into the emotional landscape of educators (Dewaele and Li, 2021; Fathi et al., 2023a; Frenzel et al., 2016). Early studies focused on the emotional dimensions of teaching, highlighting the association between positive emotions, satisfaction, and the teaching profession’s intrinsic worth (Brouwers and Tomic, 2000), progressively acknowledging their substantial impact on teacher motivation, job contentment, and overall well-being (MacIntyre and Mercer, 2014; Skaalvik and Skaalvik, 2007; Zhang et al., 2023b).

Teaching enjoyment is demonstrably linked to a related concept: foreign language enjoyment (FLE) experienced by students (Dewaele et al., 2023; Fathi et al., 2023b). FLE refers to the positive emotions and overall enjoyment students associate with learning a foreign language (Hwang et al., 2024; Liu et al., 2024). Studies reveal that teachers who exude high levels of enjoyment can foster a more positive learning environment, which in turn contributes to students’ own sense of FLE (Wu et al., 2023; Xie et al., 2023). This phenomenon, termed “emotional contagion” (Xie et al., 2023), highlights the crucial role teachers play in shaping students’ attitudes toward language learning. Furthermore, Liu et al. (2023) propose a model demonstrating the positive influence of teaching enjoyment on aspects like student grit and language achievement, further emphasizing the interconnectedness of these constructs.

Teaching enjoyment assumes profound significance within the educational sphere, exerting an influence across various facets of educators’ professional and personal lives, student outcomes, and the overall educational quality. Firstly, it stands as a cornerstone of teacher well-being, correlating with heightened job satisfaction, positive affect, and psychological well-being among educators (Skaalvik and Skaalvik, 2017; Wei et al., 2023). This sense of gratification in teaching contributes significantly to one’s professional fulfillment and sense of accomplishment within the educational sphere (Skaalvik and Skaalvik, 2010). Moreover, it aligns closely with job satisfaction, fostering increased motivation and commitment among teachers (Klassen and Chiu, 2010), thereby potentially curbing attrition rates within the profession (Ingersoll and May, 2012).

Beyond personal satisfaction, teaching enjoyment plays a key role in influencing student engagement and learning outcomes. When educators find delight in their work, their enthusiasm and motivation in the classroom become palpable, influencing and enhancing the motivation and engagement levels of students (Ruzek et al., 2016; Frenzel et al., 2009). Additionally, it fosters more effective teaching practices, with educators investing more effort in planning and implementing innovative teaching methods that invariably enhance student learning (Keller et al., 2014; Kunter et al., 2008).

Moreover, teaching enjoyment operates as a shield against teacher burnout, serving as a protective factor against emotional exhaustion and depersonalization—the core dimensions of burnout (Bakker et al., 2006; Kunter et al., 2011). The emotional resources derived from teaching enjoyment equip educators with the tools to navigate the demands and stressors inherent in teaching (Xanthopoulou et al., 2007). Organizational benefits abound as well, with educational institutions fostering a more positive school climate, enhanced teacher collaboration, and elevated staff morale in environments where teaching enjoyment is prevalent (Day et al., 2006). This uplifting environment, in turn, correlates with heightened student achievement and improved school performance. Understanding teaching enjoyment is essential in educational research and practice, as it provides key insights into improving teacher well-being, job satisfaction, and instructional quality. Contributing factors include instructional autonomy, institutional support, and individual traits such as proactive personality (Dewaele et al., 2019; Kunter et al., 2008; Xiao et al., 2022). Efforts to foster teaching enjoyment often focus on reducing job-related stress, enhancing motivation, and creating supportive environments. Recognizing the importance of teaching enjoyment has led to programs and interventions aimed at enriching educators’ positive emotional experiences, benefiting teachers, students, and the educational system as a whole.

Research has extensively examined the link between teaching enjoyment and burnout. For instance, Xiao et al. (2022) modeled the relationships between teaching enjoyment, teacher self-efficacy, and work engagement, showing their central roles in teacher well-being and satisfaction. Atmaca et al. (2020) highlighted the emotional facets of teaching and their impact on burnout and job satisfaction, while Taxer et al. (2019) identified how strong teacher-student relationships can mitigate emotional exhaustion and reduce burnout. Khajavy et al. (2017) validated a burnout model among EFL teachers, focusing on how affective and motivational factors shape burnout in this field. Together, these studies underscore the importance of teaching enjoyment as a potential factor in preventing burnout, emphasizing the need to address the emotional, motivational, and relational aspects of teaching to support educators’ job satisfaction and resilience.

### The hypothesized model

The theoretical underpinnings of this study are rooted in established literature exploring the intricate dynamics between proactive personality traits, teaching enjoyment, and teacher burnout. Building upon prior research, this study formulates and investigates three hypotheses:

*H1*: Proactive Personality and Teacher Burnout.

The first hypothesis (H1) posits a direct and negative relationship between proactive personality and teacher burnout. This hypothesis is grounded in a wealth of empirical evidence highlighting the significance of proactive traits in shaping individuals’ adaptive behaviors and responses within professional settings (Crant, 2000; Fuller and Marler, 2009). Proactive personality is characterized by an individual’s proactive and self-initiated approach to tasks, coupled with a strong inclination toward problem-solving and proactive behavior (Seibert et al., 1999). Extensive research across diverse occupational domains has consistently linked proactive personality with favorable outcomes, including heightened resilience, adaptability, and effective stress management (Pietarinen et al., 2021; Xanthopoulou et al., 2009).

The Conservation of Resources (COR) theory (Hobfoll, 1989) provides a theoretical framework to understand this relationship. According to COR theory, individuals strive to acquire and conserve resources to prevent resource depletion. Proactive individuals, equipped with personal resources such as resilience and self-efficacy, actively employ proactive strategies to prevent resource loss, including emotional exhaustion, depersonalization, and reduced personal accomplishment—the core dimensions of burnout (Hakanen and Schaufeli, 2012; Kong et al., 2021; Li et al., 2017; Pietarinen et al., 2021; Wang et al., 2021; Xanthopoulou et al., 2009; Zhang et al., 2022). Therefore, it is hypothesized that educators with proactive personality traits are less susceptible to burnout due to their proactive approach in acquiring and managing personal resources, thus demonstrating a negative association between proactive personality and teacher burnout.

*H2*: Proactive Personality and Teaching Enjoyment.

This hypothesis posits a direct and positive association between proactive personality and teaching enjoyment. Proactive individuals are characterized by self-starting tendencies, initiative, and a focus on improvement (Li et al., 2024). These qualities empower them to create engaging learning experiences (Bakker et al., 2006), actively seek solutions to classroom challenges (Crant, 2000), and ultimately derive greater enjoyment from their teaching practice (Xiao et al., 2022). Proactive teachers might be more likely to implement innovative teaching methods (Li et al., 2017), fostering a sense of accomplishment and satisfaction that contributes to their teaching enjoyment (Skaalvik and Skaalvik, 2017).

*H3*: Teaching Enjoyment as a Mediator.

The third hypothesis (H3) proposes that teaching enjoyment mediates the relationship between proactive personality and teacher burnout, establishing a sequential relationship of proactive personality → teaching enjoyment → teacher burnout. The conceptualization of teaching enjoyment stems from literature highlighting its pivotal role in mitigating burnout among educators (Keller et al., 2014; Skaalvik and Skaalvik, 2017). Teaching enjoyment reflects positive emotions, job satisfaction, and fulfillment experienced in the teaching profession, contributing significantly to reducing the risk of burnout (Atmaca et al., 2020; Bakker et al., 2006; Khajavy et al., 2017; Kunter et al., 2011; Taxer et al., 2019; Xiao et al., 2022).

The proposed mediation aligns with the Job Demands-Resources (JD-R) model (Demerouti et al., 2001). This model suggests that personal resources, such as proactive personality, act as buffers against job demands that could lead to burnout. Proactive individuals are more inclined to seek opportunities that resonate with their proactive nature, engage more deeply in their teaching, adapt their pedagogical methods to enhance student engagement, and foster a supportive classroom atmosphere (Verešová and Malá, 2012). These proactive behaviors, stemming from proactive personality traits, are expected to enhance teaching enjoyment, leading to reduced levels of burnout. Therefore, it is hypothesized that teaching enjoyment mediates the relationship between proactive personality and teacher burnout, indicating that proactive individuals experience greater teaching enjoyment, which, in turn, acts as a protective factor against burnout.

## Materials and methods

### Participants and procedures

A sample of 420 Chinese educators (236 females, 56.2%; 184 males, 43.8%) was recruited to participate in this study. These educators hail from primary and middle schools across various provinces in China, including Guangdong, Jiangsu, Zhejiang, Sichuan, and Shandong. The participants’ ages ranged from 26 to 50 years (*M* = 34.75, *SD* = 5.90). Teaching experience varied within the sample, with 198 educators (47.1%) possessing over 10 years of experience, and 222 (52.9%) having less than 10 years. Marital status distribution reflected further diversity, with 320 participants (76.2%) married or in long-term partnerships, and 100 (23.8%) single. Finally, classroom sizes averaged 35 students, encompassing a range of settings from bustling urban to serene suburban regions.

Data collection procedures involved established online survey administration platforms. Following ethical approval procedures from the Institutional Review Board (IRB) of Chongqing Preschool Education College, data collection involved established online survey administration platforms. With informed consent obtained, teachers were invited to participate through a link to the questionnaire distributed via school administration or relevant educational networks within each participating province. This online approach ensured efficient data collection from geographically dispersed participants. To ensure confidentiality and anonymity, participants completed the questionnaires independently within a designated time frame. The survey instruments, designed to take approximately 20–25 min, assessed proactive personality, teaching enjoyment, and burnout.

### Measures

To assess the key variables in this study, we utilized a set of validated self-report measures. Proactive personality traits were evaluated using the abbreviated version of the Proactive Personality Scale (S-PPS) developed by Seibert et al. (1999). This 10-item instrument asks participants to rate their agreement with statements on a 6-point Likert scale ranging from 1 (strongly disagree) to 6 (strongly agree). For example, one statement might be “I am constantly on the lookout for new ways to improve my life.” Notably, the Chinese adaptation of the S-PPS employed in this study was translated by Shang and Gan (2009). This adaptation demonstrated strong internal consistency within our sample, with a Cronbach’s alpha of 0.88, indicating reliable measurement of proactive personality.

Teaching enjoyment within the classroom environment was assessed through the Foreign Language Teaching Enjoyment Scale (FLTES)—a self-report questionnaire developed by Ergün and Dewaele (2021). Adapted from the original scale by Botes et al. (2021), the FLTES comprises nine items that categorize teaching enjoyment into three distinct dimensions: Personal Enjoyment (PE), Student Appreciation (SA), and Social Enjoyment (SE). Participants indicated their level of agreement with statements using a 5-point Likert scale, ranging from 1 (strongly disagree) to 5 (strongly agree). An example item is “The students are friendly.” While Ergün and Dewaele (2021) reported robust reliability for this instrument, its internal consistency in our study was even higher, with a Cronbach’s alpha of 0.91, signifying excellent internal consistency.

Finally, the teacher-adapted version of the Maslach Burnout Inventory (MBI-ES), validated and piloted by Maslach et al. (1997), was employed to gage burnout among participating educators. This 22-item self-report measure assesses three key dimensions of burnout: reduced personal accomplishment, depersonalization, and emotional exhaustion. Respondents rated each item on a seven-point Likert scale ranging from 0 (never) to 6 (every day). It’s important to note that within this inventory, burnout manifests as decreased scores in personal accomplishment alongside increased scores in depersonalization and emotional exhaustion. For example, a sample item from the MBI-ES is: “I feel tired at the end of the working day.” The MBI-ES also demonstrated good internal consistency in our study, with a Cronbach’s alpha of 0.81, indicating a reliable measure of burnout.

### Data analysis

Data analysis was conducted using SPSS (Version 26) and Amos (Version 26) with Maximum Likelihood Estimation (MLE). A rigorous protocol was followed to address data quality, including screening for outliers, handling missing values, and verifying multivariate normality. Missing data, which ranged between 0.71 and 1.32%, was minimal and found to meet the criteria for Missing Completely at Random (MCAR), as validated by Little’s MCAR test (χ^2^ = 1139.13, *p* = 0.541). To address these missing values, Expectation Maximization (EM), a robust imputation technique for Structural Equation Modeling (SEM), was applied (Kline, 2023).

Univariate outliers were identified using scatter plots and standardized *Z*-values, leading to the exclusion of eight cases. Skewness and kurtosis analyses confirmed that data normality fell within acceptable bounds (−1 to +1), following guidelines by Hoyle (1995) (see Table 1). Additionally, multivariate outliers were assessed using Mahalanobis distance, with one extreme case removed based on the critical chi-square cutoff at the 0.001 level, following Meyers et al. (2016). This process left a final sample of 413 participants.

**Table 1:** CFA results.

	χ^2^	df	χ^2^/df	*p*	CFI	RMSEA	SRMR	α
Burnout	210.567	110	1.914	<0.001	0.965	0.056	0.046	0.81
Proactive personality	115.783	60	1.930	<0.001	0.977	0.049	0.034	0.88
Teaching enjoyment	160.249	85	1.884	<0.001	0.981	0.043	0.030	0.91

Confirmatory Factor Analysis (CFA) was used to test the validity of measurement scales, resulting in strong model fit (refer to Table 1). Subsequently, Structural Equation Modeling (SEM) was implemented to examine whether teaching enjoyment mediated the relationship between proactive personality and burnout.

To evaluate model adequacy, we adhered to the established fit indices. These metrics included the χ^2^/df ratio, Goodness of Fit Index (GFI), Comparative Fit Index (CFI), Root-Mean-Square Error of Approximation (RMSEA), and Standardized Root-Mean-Square Residual (SRMR). An acceptable model fit was indicated by a χ^2^/df ratio below 3 and a non-significant *p*-value (*p* > 0.05). Additionally, GFI and CFI values exceeding 0.90, along with RMSEA and SRMR values under 0.08 and 0.10, respectively, were deemed to reflect good model fit (Hu and Bentler, 1999).

## Results

This research aimed to analyze three primary variables—burnout, proactive personality, and teaching enjoyment—comparing these dimensions across male and female educators. Male educators reported an average burnout score of 4.23 (SD = 0.75), whereas females had a slightly lower mean score of 4.05 (SD = 0.68). In terms of proactive personality, males averaged 3.51 (SD = 0.80), with females scoring higher at 3.70 (SD = 0.85). For teaching enjoyment, male participants scored 4.64 on average (SD = 0.92), while females averaged 4.35 (SD = 0.78).

Before performing statistical tests for group differences, we applied the Shapiro–Wilk test to confirm that each variable met the normality assumption. Results supported normal distribution for burnout (*p* = 0.203), proactive personality (*p* = 0.311), and teaching enjoyment (*p* = 0.176). Independent samples t-tests were then conducted to assess whether significant differences existed between male and female educators on these variables. Findings indicated no statistically significant differences between genders in burnout (*t* = −1.125, *p* = 0.282, Cohen’s d = 0.13), proactive personality (*t* = 0.734, *p* = 0.457, Cohen’s d = 0.09), or teaching enjoyment (*t* = −0.891, *p* = 0.376, Cohen’s d = 0.11). Table 2 provides a detailed breakdown of these test results, outlining gender comparisons for each variable measured.

**Table 2:** The results of the independent samples *t*-tests.

	Male	Female			
Variable	*M* (SD)	*M* (SD)	*t*-value	*p*-value	Cohen’s d
Burnout	4.23 (0.75)	4.05 (0.68)	−1.125	0.282	0.13
Proactive personality	3.51 (0.80)	3.70 (0.85)	0.734	0.457	0.09
Teaching enjoyment	4.64 (0.92)	4.35 (0.78)	−0.891	0.376	0.11

### Validity and reliability analysis

Table 1 presents the results of first-order confirmatory factor analyses (CFAs) and the reliability indices of the three measurement scales. The table showcases the outcomes of the first-order confirmatory factor analyses (CFAs) for burnout, proactive personality, and teaching enjoyment. Additionally, reliability indices (Cronbach’s alpha, *α*) for each construct are provided. The analyses demonstrated strong support for the reliability and validity of the measurement scales used in this study, as indicated by the confirmatory factor analysis results and the high reliability coefficients (α) for each construct.

Descriptive statistics and Pearson’s correlations among the studied constructs are presented in Table 3. The results indicate that proactive personality was positively correlated with teaching enjoyment (*r* = 0.63, *p* < 0.01) and negatively correlated with burnout (*r* = −0.49, *p* < 0.01). Teaching enjoyment exhibited a significant negative correlation with burnout (*r* = −0.49, *p* < 0.01).

**Table 3:** Descriptive statistics.

Constructs	Proactive personality	Teaching enjoyment	Burnout
1. Proactive personality	1		
2. Teaching enjoyment	0.63**	1	
3. Burnout	−0.49**	−0.49**	1
4. Mean	3.61	4.49	4.14
5. SD	0.83	0.84	0.72
6. Skewedness	−0.16	−0.18	0.11
7. Kurtosis	−0.09	−0.08	−0.24

*p < 0.05, **p < 0.01.

Descriptive statistics revealed the following: The mean score for proactive personality was 3.61 (SD = 0.83), for teaching enjoyment was 4.49 (SD = 0.84), and for burnout was 4.14 (SD = 0.72). Skewness values were found to be −0.16, −0.18, and 0.11 for proactive personality, teaching enjoyment, and burnout, respectively. Kurtosis values were −0.09, −0.08, and −0.24 for proactive personality, teaching enjoyment, and burnout, respectively. These findings provide initial insights into the relationships between proactive personality, teaching enjoyment, and burnout among EFL teachers.

### SEM analysis

**Figure 1:**
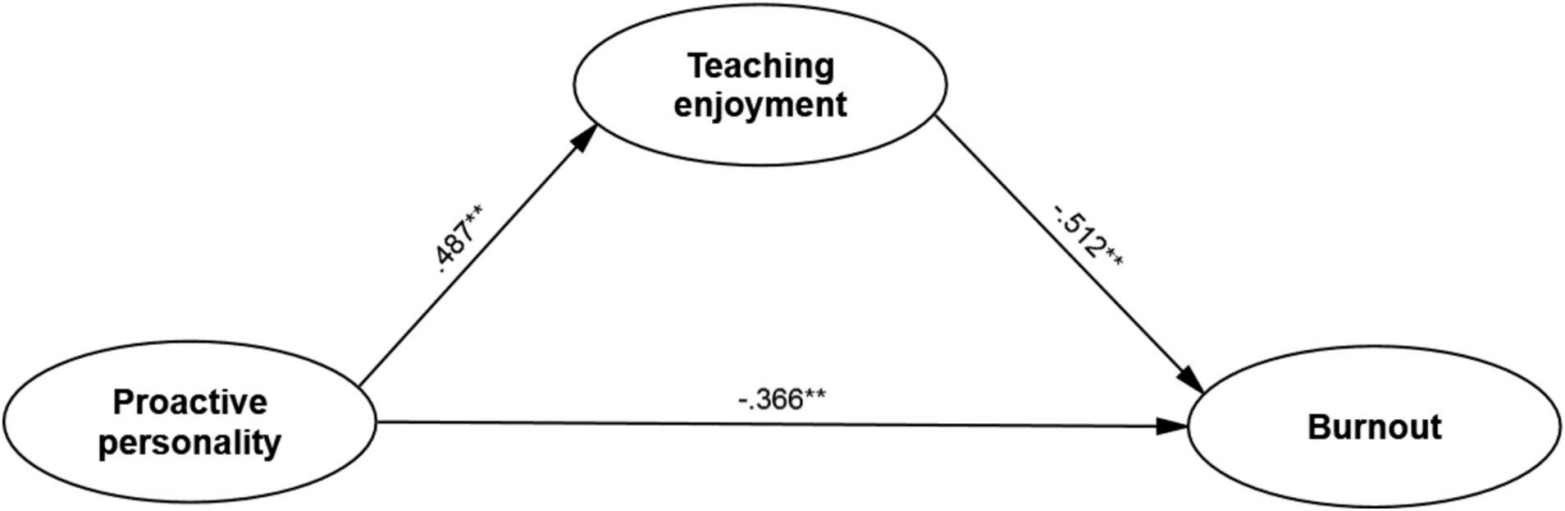
The final model of teacher burnout. All paths are significant at **p < 0.001.

Utilizing SEM, the study examined how teaching enjoyment mediates the relationship between proactive personality and teacher burnout. The structural model’s fit was evaluated through various indices for two separate groups—male and female teachers. The findings revealed a strong alignment of the proposed model with the data for male teachers (χ^2^/df = 1.620, CFI = 0.936, TLI = 0.929, IFI = 0.936, RMSEA = 0.039, and SRMR = 0.051) and female teachers (χ^2^/df = 1.543, CFI = 0.942, TLI = 0.937, IFI = 0.941, RMSEA = 0.041, and SRMR = 0.055). Figure 1 provides a visual representation of the standardized parameter estimates for this model.

To further investigate whether model coefficients differed by gender, a multi-group invariance analysis was performed. Results from the χ^2^ difference test comparing constrained and unconstrained models (Δχ^2^ = 6.283, Δdf = 5, *p* = 0.289) revealed that the mediation model’s coefficients were consistent across both male and female groups. Thus, no significant differences were identified between genders for either the direct or indirect effects of the predictor variable on the outcome variable. Additionally, to evaluate the stability of indirect effects across genders, we conducted a bootstrap resampling procedure with 500 iterations, providing further support for the robustness of the findings in both groups.

Table 4 presents the comprehensive overview of the direct and indirect effects elucidated within the structural model, uncovering the intricate interrelations among the key constructs under scrutiny. Delving into the direct effects analysis, several pivotal relationships were identified, shedding light on the associations observed within the model.

**Table 4:** SEM results of the model.

				95% CI	
Model pathways	SE	*β*	*p*	Lower bound	Upper bound
**Direct effects**					
Proactive personality → burnout	0.159	−0.366	<0.001	−0.471	−0.261
Proactive personality → Enjoyment	0.176	0.487	<0.001	0.142	0.832
Enjoyment → burnout	0.167	−0.512	<0.001	−0.645	−0.379
**Indirect effect**					
Proactive personality → enjoyment → burnout	0.102	−0.249	<0.001	−0.324	−0.073

Firstly, the path analysis revealed a significant and substantial negative relationship between proactive personality and burnout (*β* = −0.366, SE = 0.159, *p* < 0.001, 95% CI [−0.471, −0.261]). This finding underscores that higher levels of proactive personality were markedly linked to lower levels of burnout among educators, affirming the influential role of proactive tendencies in mitigating the manifestations of burnout within the teaching profession.

Moreover, a noteworthy positive relationship emerged between proactive personality and teaching enjoyment (*β* = 0.487, SE = 0.176, *p* < 0.001, 95% CI [0.142, 0.832]). This substantial positive association indicates that higher levels of proactive personality are associated with increased levels of teaching enjoyment, aligning with the notion that proactive traits may contribute to fostering a positive teaching experience. Furthermore, the analysis revealed a compelling negative relationship between teaching enjoyment and burnout (*β* = −0.512, SE = 0.167, *p* < 0.001, 95% CI [−0.645, −0.379]). This negative association suggests that higher levels of teaching enjoyment are correlated with lower levels of burnout among educators, supporting the idea that positive emotional experiences might play a protective role in mitigating burnout symptoms.

Moving to the realm of indirect effects, the investigation uncovered a significant negative indirect relationship between proactive personality and burnout, mediated by teaching enjoyment (*β* = −0.249, SE = 0.102, *p* < 0.001, 95% CI [−0.324, −0.073]). This finding suggests that proactive personality may be indirectly associated with lower levels of burnout through its positive relationship with teaching enjoyment.

### Measurement invariance analysis

To examine whether the mediation model operated equivalently across genders, we conducted a measurement invariance analysis focusing on potential differences in path coefficients between male and female teachers. The results indicated that the proposed model provided a good fit for both groups. Specifically, the multi-group invariance analysis showed acceptable fit indices for both the constrained model (χ^2^/df = 1.618, CFI = 0.932, TLI = 0.927, RMSEA = 0.038, SRMR = 0.067) and the unconstrained model (χ^2^/df = 1.617, CFI = 0.931, TLI = 0.926, RMSEA = 0.039, SRMR = 0.068).

Additionally, the chi-square difference test (Δχ^2^ = 2.715, Δdf = 3, *p* = 0.467) revealed no significant differences in the model coefficients between male and female teachers within the mediation model. This suggests that the relationships among the variables are consistent across genders. Further, we performed separate Structural Equation Modeling (SEM) analyses for male and female teachers to validate these findings. Both groups demonstrated acceptable model fits: for male teachers, the fit indices were χ^2^/df = 1.391, *p* < 0.001, CFI = 0.935, RMSEA = 0.049, SRMR = 0.068; for female teachers, the indices were χ^2^/df = 1.499, *p* < 0.001, CFI = 0.927, RMSEA = 0.052, SRMR = 0.065.

In summary, these analyses indicate that there are no significant differences between male and female teachers regarding the direct and indirect effects of proactive personality on burnout through teaching enjoyment. The mediating effect of teaching enjoyment remained stable and consistent across both genders, reinforcing the robustness of the proposed model in explaining the relationships among these variables for all participants.

## Discussion

The objective of this study was to investigate the relationship between proactive personality, teaching enjoyment, and teacher burnout within the context of educational settings. Specifically, we sought to explore how proactive personality traits among educators are related with their experiences of burnout and whether teaching enjoyment acts as a mediator in this relationship. Understanding these dynamics is pivotal in unraveling the intricate interplay between individual dispositions, emotional experiences, and occupational well-being in the realm of teaching. By examining these associations, this study aimed to contribute to the existing body of knowledge on teacher well-being and provide insights into potential mechanisms that may relate to burnout prevention, which could inform interventions designed to support educators’ professional experiences and overall job satisfaction.

First, it was found that there was a negative direct relationship observed between proactive personality and teacher burnout in our study. This aligns with and contributes to the existing literature on personality factors influencing teacher well-being and burnout (e.g., Kong et al., 2021; Li et al., 2017; Pietarinen et al., 2021; Samfira et al., 2023; Zhang et al., 2022; Wang et al., 2021). Proactive personality is associated with positive outcomes in occupational settings. Individuals with proactive personalities tend to engage in problem-solving, take initiative, and actively shape their work environments (Crant, 2000; Fuller and Marler, 2009). This disposition is linked to increased resilience, adaptability, and effective management of work-related stressors, which contrasts with the symptoms of burnout (Pietarinen et al., 2021; Seibert et al., 1999). The negative correlation between proactive personality and teacher burnout is consistent with the conservation of resources theory (Hobfoll, 1989). According to this theory, individuals strive to acquire and maintain resources to prevent their loss. Proactive individuals often possess personal resources such as resilience, self-efficacy, and adaptive coping strategies, which they use to prevent resource depletion leading to emotional exhaustion, depersonalization, and reduced personal accomplishment—the key dimensions of burnout (Hakanen and Schaufeli, 2012; Pietarinen et al., 2021; Xanthopoulou et al., 2009; Wang et al., 2021).

Moreover, the negative relationship between proactive personality and teacher burnout aligns with the broader literature on job-related stress and burnout across various professions. Studies across different occupational settings have consistently shown that proactive individuals are better equipped to manage stress and prevent burnout due to their proactive strategies for resource acquisition, utilization, and conservation (Crant, 2000; Parker et al., 2019). This resource accumulation and management may include the development of self-efficacy, a sense of control, and the ability to effectively cope with job demands (Li et al., 2017). As proactive individuals invest in these resources, they are better equipped to manage the emotional and physical demands of teaching (Van der Heijden et al., 2015), reducing their susceptibility to burnout.

This finding is also in line with the Job Demands-Resources (JD-R) model (Demerouti et al., 2001), which posits that personal resources, such as proactive personality, serve as buffers against job demands that can lead to burnout. The negative relationship between proactive personality and burnout suggests that proactive individuals may actively seek resources to manage the challenges inherent in the teaching profession. This may involve engaging in problem-focused coping strategies, seeking professional development, and adopting adaptive teaching practices that reduce the risk of burnout (Wen et al., 2022).

Furthermore, the relationship between proactive personality and teacher burnout highlights the importance of individual-level variables and support systems in educational settings (Salmela-Aro et al., 2008). Teachers with proactive personalities may benefit from programs that encourage and harness their proactive qualities, such as innovative teaching approaches, mentorship, and opportunities for professional growth (Erdogan and Bauer, 2005). These interventions can help sustain their proactive outlook and protect them from burnout. Additionally, these findings underscore the relevance of selecting and retaining educators with proactive personality traits, as they may exhibit greater resilience in the face of the inherent challenges of the teaching profession (Zhang et al., 2022).

In addition, it was found that teaching enjoyment served as a mediator in the relationship between proactive personality and teacher burnout. Again, in light of JD-R model, proactive personality can be considered a personal resource that enables individuals to effectively manage job demands. Individuals who take initiative tend to actively pursue opportunities that correspond with their personal interests, strengths, and values (Grant and Ashford, 2008; Parker et al., 2019). As a result, they may be more engaged in their teaching, adapt their pedagogical approaches to enhance student engagement, and create a positive and supportive classroom atmosphere (Verešová and Malá, 2012). These proactive behaviors appear to be linked to higher levels of teaching enjoyment, as individuals may experience greater satisfaction in their work when it is perceived as meaningful and aligns with their proactive approach (Pyhältö et al., 2021). Teaching enjoyment, in turn, acts as a protective factor against teacher burnout. Previous research has shown that higher levels of job satisfaction and positive affect are associated with lower levels of burnout (Atmaca et al., 2020; Khajavy et al., 2017; Schaufeli and Bakker, 2004; Taxer et al., 2019; Xiao et al., 2022). When teachers experience enjoyment in their work, they tend to report higher levels of satisfaction, motivation, and emotional connection to their role, which is associated with a lower likelihood of emotional exhaustion, one of the core dimensions of burnout (Maslach and Leiter, 2008).

Additionally, individuals with proactive personalities tend to actively engage in problem-solving, take initiative, and exhibit a proactive stance toward their work environment (Crant, 2000; Fuller and Marler, 2009). This proactive disposition aligns with increased resilience, adaptability, and the ability to effectively manage work-related stressors, which are antithetical to the manifestations of burnout (Pietarinen et al., 2021; Seibert et al., 1999). Also, teaching enjoyment, characterized by positive emotions, satisfaction, and fulfillment in the teaching profession, is known to play a crucial role in mitigating teacher burnout (Keller et al., 2014; Skaalvik and Skaalvik, 2017). Educators who experience high levels of teaching enjoyment are less likely to suffer from emotional exhaustion and depersonalization, the core dimensions of burnout (Bakker et al., 2006; Kunter et al., 2011). Recognizing teaching enjoyment as a mediator highlights its potential role in lessening the negative impact of occupational stressors, which may be associated with proactive personality traits and, in turn, relate to lower levels of teacher burnout. This finding resonates with previous research highlighting the protective role of positive emotions and satisfaction in reducing the risk of burnout among educators (Frondozo et al., 2020; Schaufeli et al., 2006).

Furthermore, this mediation finding is consistent with COR theory (Hobfoll, 1989), which posits that individuals strive to acquire and preserve resources to avoid depletion. Proactive individuals may possess personal resources, such as resilience, self-efficacy, and adaptive coping skills, which support their capacity to experience teaching enjoyment. In turn, teaching enjoyment functions as an emotional resource, enabling educators to manage teaching-related demands and reduce burnout risk (Xanthopoulou et al., 2007). Thus, the mediating role of teaching enjoyment in the link between proactive personality and teacher burnout underscores the significance of individual traits and emotional factors in the teaching profession. By understanding how proactive personality may be associated with teaching enjoyment and how this relationship could potentially reduce teacher burnout, educators and educational institutions can consider targeted steps to support the well-being and job satisfaction of teachers.

## Conclusion and implications

Taken together, this study explores the relationships between proactive personality, teaching enjoyment, and teacher burnout, highlighting teaching enjoyment as a potential mediator in the association between proactive personality and burnout. Findings suggest that proactive traits may support teaching enjoyment, which could help protect against burnout. These results carry practical implications for educators and educational institutions, highlighting the importance of recognizing and nurturing proactive traits in teachers, as well as fostering positive emotional experiences in the teaching profession. By examining how proactive personality correlates with burnout, educators, administrators, and policymakers can develop strategies aimed at enhancing teachers’ well-being and job satisfaction, thereby potentially improving educational outcomes.

For educators, understanding the connection between proactive personality, teaching enjoyment, and burnout provides a basis for self-reflection and professional development. Proactive individuals may benefit from tailored development opportunities that leverage their strengths, which could enrich their teaching experiences and mitigate burnout risk. For example, professional development programs focused on goal-setting, problem-solving, and innovative teaching practices may be particularly valuable for proactive teachers. Additionally, educators can cultivate proactive behaviors by taking on leadership roles within their schools or participating in curriculum development initiatives.

Educational institutions can apply these findings by creating policies that support teacher well-being. Building a collaborative work environment, providing professional development opportunities, and recognizing proactive teachers’ contributions can enhance job satisfaction and engagement. Additionally, including proactive personality assessments in recruitment and development processes could help identify candidates who are likely to thrive in teaching roles. Schools may also consider evidence-based interventions designed to increase teaching enjoyment and reduce burnout. For example, autonomy-supportive training for teachers, as suggested by Tilga et al. (2021), has been shown to improve teachers’ psychological well-being and teaching efficacy. Empowering students to take responsibility for their learning and promoting self-determination can foster a more positive classroom environment.

From a policy perspective, this study highlights the need for strategies focused on teacher well-being and job satisfaction. Investments in training, support systems, and professional development that align with proactive traits can help build a resilient teaching workforce. Recognizing teaching enjoyment as a buffer against burnout may further inform policies aimed at improving educational outcomes.

Despite these contributions, several limitations must be acknowledged. First, the sample size and demographic scope may restrict the generalizability of the findings, and future research using larger and more diverse samples could enhance external validity. Second, the cross-sectional design captures associations between variables at a single time point, which restricts our ability to draw causal conclusions. Longitudinal studies following participants over extended periods or experimental studies that manipulate variables could provide more robust insights into potential causal pathways. Additionally, the study relies on self-report measures, which can introduce response biases. Incorporating multiple data sources, such as peer or supervisor evaluations and administrative data, would add depth and strengthen the validity of the findings. Lastly, this study did not specifically examine contextual factors such as school culture, administrative support, or job demands, which may significantly impact teacher well-being. Future research should investigate these contextual influences to provide a more comprehensive understanding of factors affecting teacher burnout and job satisfaction.

## Data Availability

The data analyzed in this study is subject to the following licenses/restrictions: The raw data supporting the conclusions of this article will be made available by the authors, without undue reservation. Requests to access these datasets should be directed to Hu Chunyan, Email: Cindy_Hu202308@126.com.
